# Clinical Features of Thoracic Myelopathy: A Single-Center Study

**DOI:** 10.5435/JAAOSGlobal-D-18-00090

**Published:** 2019-11-04

**Authors:** Kei Ando, Shiro Imagama, Kazuyoshi Kobayashi, Kenyu Ito, Mikito Tsushima, Masayoshi Morozumi, Satoshi Tanaka, Masaaki Machino, Kyotaro Ota, Hiroaki Nakashima, Yoshihiro Nishida, Naoki Ishiguro

**Affiliations:** From the Department of Orthopaedic Surgery, Nagoya University Graduate School of Medicine, Nagoya, Aichi.

## Abstract

Thoracic myelopathy is relatively uncommon because few degenerative changes occur as a result of the restricted range of motion surrounding the rib cage.

**Methods:**

A retrospective study was performed in 300 consecutive surgical cases of thoracic disorders with myelopathy treated in our department from 2000 to 2015. Girdle pain, back pain, low back pain, leg numbness, leg pain, gait disturbance, leg paresis, and bowel bladder disturbance as initial and preoperative symptoms; patellar tendon reflex, ankle tendon reflex, and ankle clonus as preoperative neurologic findings; MRI and CT findings; and surgical procedure, intraoperative findings, and postoperative recovery were investigated.

**Results:**

The disease distribution included ossification of the ligamentum flavum (OLF) (n = 48), ossification of the posterior longitudinal ligament (OPLL) (n = 30), OPLL with OLF (n = 27), intradural extramedullary tumor (n = 98), intramedullary spinal cord tumor (n = 64), vertebral tumor (n = 17), spinal cord herniation (n = 7), vertebral fracture (n = 4), and thoracic disk herniation (n = 5). There were notable associations of gait disturbance with OPLL and OPLL + OLF; back pain at initial diagnosis with disease at upper levels; and low back pain with disease at a lower level.

**Conclusion:**

These findings suggest that patients with gait disturbance, back pain, and low back pain on physical examination may have thoracic disease that results in myelopathy.

Thoracic myelopathy is relatively uncommon because few degenerative changes occur as a result of the restricted range of motion surrounding the rib cage. Disorders that can cause thoracic myelopathy include ossification of the posterior longitudinal ligament (OPLL), ossification of the ligamentum flavum (OLF), spinal tumor, spinal cord tumor, trauma, infection, thoracic disk disease, and spinal cord herniation (SCH).^1–3^ These disorders all generally require surgical intervention because of their progressive nature and poor response to conservative therapy.^4,5^

Thoracic myelopathy can cause symptoms such as leg pain, girdle pain, back pain, low back pain, motor and sensory deficit, and bowel bladder dysfunction. However, these symptoms mimic those of cervical or lumbar spinal disorders, and thus, thoracic myelopathy is often overlooked in a search for a cervical or lumbar etiology, which can result in delayed treatment.^6,7^ Misdiagnosis can also lead to a prolonged preoperative disease duration, which can result in myelopathy and cause irreversible neurologic damage.^2^ However, the characteristics of thoracic myelopathy have not been adequately addressed because of the low prevalence and the small number of patients treated surgically.^2^

Knowledge of the symptoms of thoracic myelopathy is important for early diagnosis and treatment. Therefore, in this study, we examined symptoms and physical examination findings associated with diseases that cause myelopathy at each level of the thoracic spine, with the goal of identifying factors that are associated with these diseases.

## Methods

A retrospective study was performed in 300 consecutive cases of thoracic disorders with myelopathy treated at our department from 2000 to 2015 (Table 1). The study was approved by the institutional review board of our university.

**Table 1:** Summary of Demographic Data in 300 Patients With Thoracic Myelopathy

Sex	
Male	166
Female	134
Age (yr)	54.5 ± 16.2 (13-86)
Disease duration (mo)	17.0 ± 32.5 (0.5-120)
Preoperative JOA score	5.7 ± 2.8 (0-9)
Postoperative JOA score	7.5 ± 2.2 (0-11)
JOA recovery rate	38.2 ± 51.6 (−75-100)

JOA = Japanese Orthopaedic Association

Data are shown as mean ± SD with range in parentheses.

The severity of myelopathy before and after surgery was evaluated using the Japanese Orthopaedic Association (JOA) score for thoracic myelopathy (total of 11 points), which was derived from the JOA score for cervical myelopathy by eliminating the motor and sensory scores for the upper extremities.^8,9^ Postoperative improvement of symptoms was evaluated using the % recovery of the JOA score and the Hirabayashi method ([postoperative JOA score − preoperative JOA score]/[11 − preoperative JOA score] × 100%), with a recovery rate of 100% indicating the best postoperative improvement.

Girdle pain, back pain, low back pain, leg numbness, leg pain, gait disturbance, leg paresis, and bowel bladder disturbance (BBD) as initial and preoperative symptoms; patellar tendon reflex (PTR), ankle tendon reflex (ATR), and ankle clonus as preoperative neurologic findings; MRI and CT findings; and surgical procedure, intraoperative findings, and postoperative recovery were investigated. T1-4 was defined as the upper level, T5-8 as the middle level, and T9-12 as the lower level. The follow-up period was a minimum of 1 year.

Data were analyzed using SPSS ver. 22 (IBM SPSS Statistics 19.0; IBM). Data are presented as mean ± SD. Radiographic parameters and clinical data from 2 groups were compared by the Student *t*-test. Multivariate logistic regression analyses were used to calculate the odds ratios (ORs) and 95% confidence interval (CI) to identify factors associated with disease or affected level. *P* < 0.05 was considered significant.

## Results

### Clinical Manifestation

Sex, mean age at surgery, disease duration, follow-up period, preoperative and postoperative JOA score, JOA recovery rate, disease distribution, affected level, initial and preoperative symptoms, and neurologic findings are described in Tables 1 and 2. The 300 patients (166 men and 134 women) had an age range of 13 to 86 years (mean age, 54.7 years). The mean disease duration from onset to surgery was 17.0 months (range, 0.5 to 120 months). The diseases included OLF (n = 48), OPLL (n = 30), OPLL with OLF (n = 27), intradural extramedullary tumor (n = 98), intramedullary spinal cord tumor (IMSCT) (n = 64), vertebral tumor (VT) (n = 17), SCH (n = 7), vertebral fracture (VF) (n = 4), and thoracic disk herniation (TDH) (n = 5). The number of symptoms just before surgery was higher than the number of initial symptoms. Moreover, the rate of myelopathy such as leg numbness, gait disturbance, leg paresis, and BBD notably increased more than that of girdle pain, back pain, and low back pain (Table 2). There was a significant improvement in the mean JOA score from 5.7 preoperatively to 7.5 at the last follow-up postoperatively (*P* < 0.01), and this change gave a mean recovery rate of 38.2% (Table 1).

**Table 2:** Clinical Features of Patients With Thoracic Myelopathy

Disease	
OLF	48 (16%)
OPLL	30 (10%)
OPLL + OLF	27 (9%)
IET	98 (32.7%)
IMSCT	64 (21.3%)
VT	17 (5.7%)
SCH	7 (2.3%)
VF	4 (1.3%)
TDH	5 (1.7%)
Affected level	
Upper	68 (22.6%)
Middle	103 (34.2%)
Lower	129 (42.9%)
Initial/preoperative symptoms	
Girdle pain	19 (6.3%)/22 (7.3%)
Back pain	49 (16.3%)/58 (19.3%)
Low back pain	66 (21.9%)/82 (27.2%)
Leg numbness	159 (52.8%)/242 (80.2%)
Leg pain	49 (16.3%)/66 (21.9%)
Gait disturbance	78 (25.9%)/193 (64.1%)
Leg paresis	21 (7%)/102 (33.9%)
BBD	9 (3%)/27 (9%)
Neurologic findings	
PTR	196 (65.1%)
ATR	179 (59.5%)
Ankle clonus	122 (40.5%)

ATR = ankle tendon reflex, BBD = bowel bladder disturbance, IET = intradural extramedullary tumor, IMSCT = intramedullary spinal cord tumor, OLF = ossification of the ligamentum flavum, OPLL = ossification of the posterior longitudinal ligament, PTR = patellar tendon reflex, SCH = spinal cord herniation, TDH = thoracic disk herniation, VF = vertebral fracture, VT = vertebral tumor

### Disease and Affected Level

Sex, age, and rates of hyper-PTR and hyper-ATR were similar among the disease groups. However, the disease duration tended to be longer for IMSCT and SCH and shorter for VT, VF, and TDH compared with other diseases. The preoperative JOA score was lower in OPLL + OLF and SCH. OLF occurred at a lower level, and OPLL and OPLL + OLF were found at a middle level most frequently. The JOA recovery rate was lower in OPLL, OPLL + OLF, and IMSCT (Table 3). For the affected levels, sex, age, disease duration, and preoperative and postoperative JOA scores were similar, but hyper-PTR, hyper-ATR, and positive ankle clonus occurred less frequently at the lower level (Table 4).

**Table 3:** Patients' Demographics for Each Disease

Factor	OLF	OPLL	OPLL + OLF	IET	IMSCT	VT	SCH	VF	TDH
n	48	30	27	98	64	17	7	4	5
Sex									
Male	35 (72.9%)	17 (56.7%)	14 (51.9%)	51 (52.0%)	36 (56.3%)	9 (52.9%)	2 (28.6%)	1 (25.0%)	2 (40.0%)
Female	13 (27.1%)	13 (43.3%)	13 (48.1%)	47 (48.0%)	28 (43.8%)	8 (47.1%)	5 (71.4%)	3 (75.0%)	3 (60.0%)
Mean age (years)	56.5 ± 15.6 yr	52.3 ± 14.8 yr	52.8 ± 10.4 yr	54.9 ± 16.4 yr	44.8 ± 16.4 yr	52.3 ± 18.5 yr	54.1 ± 9.4 yr	74.3 ± 6.4 yr	54.2 ± 12.6 yr
Mean disease duration (mo)	17.4 ± 18.8 mo	10.0 ± 9.6 mo	17.4 ± 26.5 mo	19.0 ± 40.7 mo	21.3 ± 40.0 mo	7.1 ± 14.1 mo	24.4 ± 42.3 mo	5.0 ± 3.6 mo	4.4 ± 3.5 mo
Preoperative JOA score	6.2 ± 2.5	4.8 ± 2.5	3.9 ± 2.4	6.4 ± 42.8	6.2 ± 2.6	4.1 ± 2.9	3.4 ± 1.5	5.3 ± 2.6	5.8 ± 3.9
Postoperative JOA score	8.2 ± 1.9	6.8 ± 2.0	6.2 ± 1.9	8.1 ± 2.1	7.3 ± 2.4	6.8 ± 1.8	5.9 ± 2.0	7.3 ± 2.9	7.0 ± 3.5
JOA recovery rate (%)	45.8 ± 21.6	35.5 ± 17.2	34.3 ± 18.7	42.1 ± 28.0	29.3 ± 33.0	36.5 ± 18.2	32.4 ± 18.5	47.2 ± 37.4	38.2 ± 35.3
Affected level									
Upper	7 (14.6%)	8 (26.7%)	5 (18.5%)	24 (24.5%)	16 (25.0%)	7 (41.2%)	2 (28.6%)	0 (0.0%)	0 (0.0%)
Middle	3 (6.3%)	19 (63.3%)	18 (66.7%)	29 (29.6%)	16 (25.0%)	7 (41.2%)	5 (71.4%)	1 (25.0%)	3 (60.0%)
Lower	38 (79.2%)	3 (10.0%)	3 (11.1%)	45 (45.9%)	32 (50.0%)	3 (17.6%)	0 (0.0%)	3 (75.0%)	2 (40.0%)
Initial/preoperative symptoms									
Girdle pain	0 (0%)/0 (0%)	2 (6.7%)/2 (6.7%)	1 (3.7%)/4 (4.1%)	4 (4.1%)/2 (7.4%)	7 (10.9%)/8 (12.5%)	5 (29.4%)/6 (35.3%)	0 (0.0%)/0 (0.0%)	0 (0.0%)/0 (0.0%)	0 (0.0%)/0 (0.0%)
Back pain	0 (0%)/2 (4.2%)	3 (10.0%)/3 (10.2%)	3 (11.1%)/3 (11.1%)	17 (17.3%)/20 (20.4%)	16 (25.0%)/17 (26.6%)	7 (41.2%)/10 (58.8%)	0 (0.0%)/0 (0.0%)	2 (50.0%)/2 (50.0%)	0 (0.0%)/0 (0.0%)
Low back pain	8 (16.7%)/12 (25.0%)	4 (13.3%)/6 (20.0%)	2 (7.4%)/3 (11.1%)	29 (29.6%)/34 (34.7%)	16 (25.0%)/19 (29.7%)	3 (17.6%)/3 (17.6%)	1 (14.3%)/2 (28.6%)	2 (50.0%)/2 (50.0%)	2 (40.0%)/2 (40.0%)
Leg numbness	32 (66.7%)/33 (68.8%)	18 (60.0%)/23 (76.7%)	17 (63.0%)/21 (77.8%)	45 (45.9%)/63 (64.3%)	36 (56.3%)/50 (78.1%)	6 (35.3%)/10 (58.8%)	4 (57.1%)/4 (57.1%)	0 (0.0%)/3 (25.0%)	1 (20.0%)/2 (40.0%)
Leg pain	8 (16.7%)/10 (20.8%)	4 (13.3%)/7 (23.3%)	2 (7.4%)/4 (14.8%)	22 (22.4%)/28 (28.6%)	10 (15.6%)/12 (18.8%)	2 (11.8%)/3 (17.6%)	1 (14.3%)/1 (14.3%)	0 (0.0%)/0 (0.0%)	1 (20.0%)/3 (60.0%)
Gait disturbance	17 (35.4%)/31 (64.6%)	14 (46.7%)/27 (90.6%)	14 (51.9%)/23 (85.2)%	17 (17.3%)/48 (49.0)%	9 (14.1%)/35 (54.7)%	3 (17.6%)/15 (88.2)%	1 (14.3%)/7 (100%)	0 (0.0%)/3 (25.0%)	2 (40.0%)/3 (60.0)%
Leg paresis	5 (10.4%)/10 (20.8%)	2 (6.7%)/13 (43.3%)	3 (11.1%)/11 (40.7%)	4 (4.1%)/28 (28.6%)	4 (6.3%)/17 (26.6%)	1 (5.9%)/12 (70.6%)	0 (0.0%)/3 (42.9%)	0 (0.0%)/3 (25.0%)	2 (40.0%)/3 (60.0)%
BBD	1 (2.1%)/2 (4.2%)	2 (6.7%)/4 (13.3%)	0 (0.0%)/1 (3.7%)	1 (1.0%)/9 (9.2%)	3 (4.7%)/6 (9.4%)	2 (11.8%)/4 (23.5%)	0 (0.0%)/0 (0.0%)	0 (0.0%)/0 (0.0%)	0 (0.0%)/0 (0.0%)
Neurologic findings									
Hyper-PTR	29 (60.4%)	27 (90.0%)	24 (88.9%)	48 (49.0%)	41 (64.1%)	13 (76.5%)	6 (85.7%)	3 (75.0%)	4 (80.0%)
Hyper-ATR	25 (52.1%)	22 (73.3%)	21 (77.8%)	43 (43.9%)	41 (64.1%)	15 (88.2%)	5 (71.4%)	2 (50.0%)	4 (80.0%)
Positive ankle clonus	16 (33.3%)	20 (66.7%)	20 (74.1%)	28 (28.6%)	24 (37.5%)	9 (52.9%)	3 (42.9%)	0 (0.0%)	4 (80.0%)

ATR = ankle tendon reflex, BBD = bowel bladder disturbance, IET = intradural extramedullary tumor, IMSCT = intramedullary spinal cord tumor, JOA = Japanese Orthopaedic Association, OLF = ossification of the ligamentum flavum, OPLL = ossification of the posterior longitudinal ligament, PTR = patellar tendon reflex, SCH = spinal cord herniation, TDH = thoracic disk herniation, VF = vertebral fracture, VT = vertebral tumor

**Table 4:** Patients' Demographics for Each Affected Level

Factor	Upper	Middle	Lower
n	69	102	128
Sex			
Male	42 (60.9%)	55 (53.9%)	69 (53.9%)
Female	27 (39.1%)	47 (46.1%)	59 (46.1%)
Mean age (yr)	51.5 ± 15.2 yr	52.2 ± 15.7 yr	53.3 ± 17.1 yr
Preoperative JOA score	5.4 ± 2.9	4.9 ± 2.6	17.3 ± 35.9 mo
Mean disease duration (mo)	20.0 ± 39.2 mo	13.6 ± 20.6 mo	6.6 ± 2.5
Postoperative JOA score	7.4 ± 2.3	6.9 ± 2.1	8.1 ± 2.2
JOA recovery rate (%)	41.8 ± 27.0	34.1 ± 19.7	39.3 ± 30.7
Initial/preoperative symptoms			
Girdle pain	12 (17.4%)/15 (21.7%)	7 (6.9%)/7 (6.9%)	0 (0.0%)/0 (0.0%)
Back pain	21 (30.4%)/28 (40.6%)	24 (23.5%)/26 (25.5%)	4 (3.1%)/4 (3.1%)
Low back pain	4 (5.8%)/8 (11.6%)	9 (8.8%)/12 (11.8%)	53 (41.4%)/62 (48.4%)
Leg numbness	39 (56.5%)/49 (71.0%)	55 (53.9%)/75 (73.5%)	64 (50.0%)/85 (66.4%)
Leg pain	7 (10.1%)/9 (13.0%)	12 (11.8%)/17 (16.7%)	29 (22.7%)/39 (30.5%)
Gait disturbance	17 (24.6%)/44 (63.8%)	29 (28.4%)/79 (77.5%)	31 (24.2%)/68 (53.1%)
Leg paresis	3 (4.3%)/16 (23.2%)	7 (6.9%)/53 (52.0%)	11 (8.6%)/32 (25.0%)
BBD	1 (1.4%)/4 (5.8%)	3 (2.9%)/13 (12.7%)	5 (3.9%)/9 (7.0%)
Neurologic findings			
Hyper-PTR	56 (81.2%)	82 (80.4%)	57 (44.5%)
Hyper-ATR	51 (73.9%)	74 (72.5%)	53 (41.4%)
Positive ankle clonus	38 (55.1%)	53 (52.0%)	30 (23.4%)
Disease			
OLF	7 (10.1%)	3 (2.9%)	38 (29.7%)
OPLL	8 (11.6%)	19 (18.6%)	3 (2.3%)
OPLL + OLF	5 (7.2%)	18 (17.6%)	3 (2.3%)
IET	24 (34.8%)	29 (28.4%)	45 (35.2%)
IMSCT	16 (23.2%)	16 (15.7%)	32 (25.0%)
VT	7 (10.1%)	7 (6.9%)	3 (2.3%)
SCH	2 (2.9%)	5 (4.9%)	0 (0.0%)
VF	0 (0.0%)	1 (1.0%)	3 (2.3%)
TDH	0 (0.0%)	3 (2.9%)	2 (1.6%)

ATR = ankle tendon reflex, BBD = bowel bladder disturbance, IET = intradural extramedullary tumor, IMSCT = intramedullary spinal cord tumor, JOA = Japanese Orthopaedic Association, OLF = ossification of the ligamentum flavum, OPLL = ossification of the posterior longitudinal ligament, PTR = patellar tendon reflex, SCH = spinal cord herniation, TDH = thoracic disk herniation, VF = vertebral fracture, VT = vertebral tumor

### Factors Related to Disease and Affected Level

**Figure 1:**
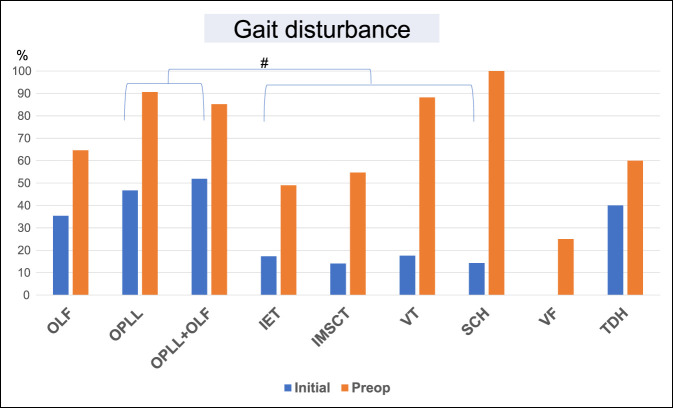
Bar graph showing that there were notable associations of gait disturbance at initial diagnosis with OPLL and OPLL + OLF; #*P* < 0.01. IET = intradural extramedullary tumor, IMSCT = intramedullary spinal cord tumor, OLF = ossification of the ligamentum flavum, OPLL = ossification of the posterior longitudinal ligament, SCH = spinal cord herniation, TDH = thoracic disk herniation, VF = vertebral fracture, VT = vertebral tumor

**Figure 2:**
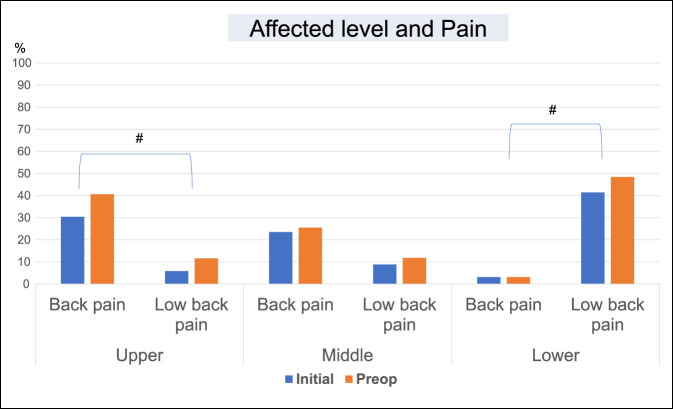
Bar graph showing that back pain at initial diagnosis with disease at a upper level and low back pain with disease at a lower level were significantly higher than another pain; #*P* < 0.01.

Multivariate analyses were performed using the above variables to identify factors that were notably related to disease and affected level. In these analyses, there were significant associations of gait disturbance at initial diagnosis with OPLL (OR, 2.74, 95% CI, 1.16 to 6.50, *P* = 0.022) and OPLL + OLF (OR, 4.18, 95% CI, 1.66 to 10.53, *P* = 0.002) (Table 5 and Figure 1); back pain at initial diagnosis with disease at upper (OR, 2.31, 95% CI, 1.14 to 4.66, *P* = 0.02) and middle (OR, 2.22, 95% CI, 1.13 to 4.39, *P* = 0.02) levels; and low back pain with disease at a lower level (OR, 5.12, 95% CI, 2.47 to 10.60, *P* < 0.01) (Table 6 and Figure 2).

**Table 5:** Factors Related to Disease Based on Multiple Logistic Regression Analysis

Disease		Odds Ratio (95% Confidence Interval)	*P*
OPLL	Gait disturbance (initial)	2.74 (1.16-6.50)	0.022
OPLL + OLF	Gait disturbance (initial)	4.18 (1.66-10.53)	0.002

OLF = ossification of the ligamentum flavum, OPLL = ossification of the posterior longitudinal ligament

**Table 6:** Factors Related to Affected Level Based on Multiple Logistic Regression Analysis

Affected level		Odds Ratio (95% Confidence Interval)	*P*
Upper	Back pain (initial)	2.31 (1.14-4.66)	0.02
Middle	Back pain (initial)	2.22 (1.13-4.39)	0.02
Lower	Low back pain (initial)	5.12 (2.47-10.60)	<0.01

## Discussion

Thoracic disease with myelopathy is relatively rare, and diagnosis may be delayed.^10^ The disease can be diagnosed by MRI and CT,^11^ but criteria are based on limited reports with relatively few patients with thoracic ossification, spondylosis, and disk herniation.^1,2^ In this study, we included other disorders that are causes of thoracic myelopathy, including spinal tumor, spinal cord tumor, trauma, ossification of the ligament, and SCH, in addition to these diseases, and we identified factors associated with these diseases by comparing symptoms and physical examination findings.

The mean disease duration of IMSCT and SCH tended to be longer than that for other diseases. These diseases are rare,^12–14^ and the period until surgery may reflect the longer time required for diagnosis. Similarly, the shorter disease durations of VT, VF, and TDH may be linked to their relative ease of diagnosis using radiograph, MRI, and CT.^15,16^ As in a previous report, OLF occurred at lower levels of the thoracic spine and OPLL and OPLL + OLF at middle levels.^17–19^ The reasons for the high frequency of OLF at lower thoracic levels include increased mechanical stress where the thoracic vertebrae form the junction between the rigid rib cage and elastic lumbar spine, a direct correlation between increased mobility of the spine and repetitive mild trauma, and high tensile force present in the posterior column.^17^ Matsumoto et al^20^ analyzed the surgical outcomes of patients with thoracic OPLL and found an average recovery rate of 36% and reduction of myelopathy immediately after surgery in 12% of patients. As preoperative myelopathy became severe, the JOA recovery rate became worse.

It is generally considered that PTR and ATR were hyper in patients with myelopathy. However, hyper-PTR, hyper-ATR, and positive ankle clonus occurred less frequently at the lower level in this study. The low rate of hyper-PTR, hyper-ATR, and positive ankle clonus may be explained by the involvement of conus and lumbar nerve roots.^21^ We should be aware of the possibility of lower thoracic diseases without hyper-PTR and hyper-ATR in thoracic myelopathy.

The number of symptoms just before surgery was higher than the number of initial symptoms. Moreover, an increase in the rate of change in preoperative symptoms compared with initial symptoms such as leg numbness, gait disturbance, leg paresis, and BBD was notably higher than that of girdle pain, back pain, and low back pain. In other words, it might mean the finding of increased neurologic compromise with no clear increase in the pain level at the time of surgery compared with the time of the initial diagnosis.

Gait disturbance at initial diagnosis, including leg palsy, leg numbness, posterior column ataxia, and spasticity, was notably associated with OPLL and OPLL + OLF. These are generally severe conditions because of spinal cord compression from the anterior or posterior side, which results in various symptoms. Gait disturbance is a representative symptom for patients with thoracic ossification.^22^ Back pain at initial diagnosis was notably associated with disease at the upper and middle levels, whereas low back pain was linked to disease at the lower level. The high prevalence of low back pain in patients with lower level disease may be explained by the involvement of the conus, which is a target for treatment of low back pain.^23,24^ Several case reports have indicated a relationship of back pain with upper and middle level disorders,^25,26^ and we speculate that a change in pressure in the spinal cord and vertebral column could lead to back pain.

To the best of our knowledge, this is the first report to examine factors related to diseases and levels that result in thoracic myelopathy. It was suggested that the increased neurologic compromise at the time of surgery compared with the time of the initial diagnosis was not associated with the pain level. Gait disturbance at initial diagnosis was associated with OPLL and OPLL + OLF. Back pain at initial diagnosis was associated with disease at the upper and middle levels, whereas low back pain was linked to disease at the lower level.

## Conclusion

We examined the characteristic factors related to diseases at each level of the thoracic spine that can result in myelopathy, based on symptoms and physical examination findings. We should be aware of the possibility of lower thoracic diseases without hyper-PTR and hyper-ATR in thoracic myelopathy. The increased neurologic compromise at the time of surgery compared with the time of the initial diagnosis was not associated with the pain level. OPLL and OPLL + OLF were characterized by gait disturbance at initial diagnosis. Back pain at initial diagnosis was associated with upper and middle level disease, and low back pain was linked to lower level disease.
